# The Use of Zinc Gelatine

**Published:** 1902-10-11

**Authors:** 


### THE USE OF ZINC GELATINE.

Dr. Alfred Eddowes,[1] in describing the advantages of the gelatine dressings introduced by Unna, insists upon the necessity of their being properly applied, and more especially of the skin being efficiently prepared so as to provide against the rancidity of the fatty secretions as well as the chemical changes in the sweat and débris of dead epithelial cells, which are likely to occur if we content ourselves with painting the gelatine on a greasy, dirty (*i.e.* septic) surface. The skin is first to be cleansed by wetting and gently wiping it with spirit of wine, and while this is evaporating it is to be dusted with a powder consisting of four parts of starch and one of calomel. The calomel is a permanent, non-irritating, and rather insoluble antiseptic, which serves better for this purpose than anything else. Time is to be given for the spirit to thoroughly dry up or soak in before the gelatine is painted on, for if this comes in contact with the spirit it will become hardened and stiffened, and will not adhere to the skin. The formula which Dr. Eddowes employs is as follows: Zinc oxide, gelatine, glycerine, and water, in the proportions of one, two, three, and four in the order mentioned. The gelatine is soaked for a few hours in part of the water, and then all the ingredients are mixed and managed afterwards precisely as glue. When freshly made the above proportions produce a mixture of the right consistency, but when it has been kept for many weeks, and especially if it has been heated several times over, it will require a little water to be added from time to time to keep it sufficiently thin for use. It is to be heated in, and used directly from, an ordinary glue-pot.

[1] The Medical Times, Sept. 27.

